# The Potential for Twice‐Annual Influenza Vaccination to Reduce Disease Burden

**DOI:** 10.1111/irv.70052

**Published:** 2025-03-06

**Authors:** Shuyi Zhong, Mark G. Thompson, Benjamin J. Cowling

**Affiliations:** ^1^ School of Public Health University of Hong Kong Hong Kong China; ^2^ Westat Rockville Maryland USA; ^3^ Laboratory of Data Discovery for Health Limited Hong Kong Science and Technology Park Hong Kong China

**Keywords:** influenza vaccination, public health, vaccine effectiveness

## Abstract

**Background:**

Influenza vaccination is recommended annually based on the evolving nature of influenza viruses and the waning of vaccine‐induced immunity. The timing of vaccination is usually before the winter influenza season in most temperate locations, where the seasonality is clear and influenza activities on average last no longer than 6 months. However, many tropical and subtropical areas have year‐round influenza activity and multiple epidemics within 1 year, against which annual influenza vaccination may not offer sufficient protection at the individual level.

**Aims:**

A twice‐annual vaccination program could utilize standard inactivated influenza vaccines or enhanced influenza vaccines. Here, we discuss three reasons to consider twice‐annual vaccination as a strategy to improve protection.

**Discussion:**

The first, mentioned above, is that some locations experience prolonged or year‐round influenza activity. The second reason is based on the observation that vaccine effectiveness significantly declines about 6 months after vaccination particularly for A(H3N2) strains, and therefore, vaccination twice a year might be beneficial to maintain a higher level of immunity in the second half of each year. The third reason is to allow for receipt of the most updated vaccine strains, given that these are updated twice each year by the World Health Organization. We also discuss three potential barriers or challenges. The first potential challenge is knowledge gaps, because there are very few existing studies that used twice‐annual vaccination. The second potential barrier is a concern over whether more frequent vaccination would lead to reduced immunogenicity or reduced clinical protection in the longer term. The third relates to concerns about cost or feasibility.

**Conclusion:**

We discuss these issues and recommend comparative assessment of the incremental benefits and cost of twice‐annual vaccination versus annual vaccination, as well as other vaccination strategies aiming to reduce influenza disease burden particularly in tropical and subtropical locations where there can be year‐round influenza activity.

## 1 Introduction

The World Health Organization (WHO) recommends annual influenza vaccination for all individuals > 6 months of age [1]. Whereas some locations have been able to achieve high coverage of influenza vaccination annually, particularly in targeted groups such as older adults, opportunities remain for further reductions in influenza disease burden [2, 3]. In recent years, the introduction of enhanced influenza vaccines provide an opportunity to improve protection for older adults [4, 5, 6]. Another option, which has not been given as much consideration, is increasing the vaccination frequency to twice per year instead of once per year with conventional “standard‐dose” inactivated influenza vaccines that include 15‐μg hemagglutinin per strain. Twice‐annual vaccination strategies could also be considered with enhanced influenza vaccines. Here, we summarize the considerations and evidence available for or against a twice‐annual influenza vaccination strategy with standard‐dose vaccines at individual level. We first give three reasons to consider a twice‐annual vaccination strategy, we then discuss three potential obstacles, and we end with some conclusions and recommendations.

## 2 Rationale for Twice‐Annual Vaccination

### 2.1 Influenza Seasonality

**FIGURE 1:**
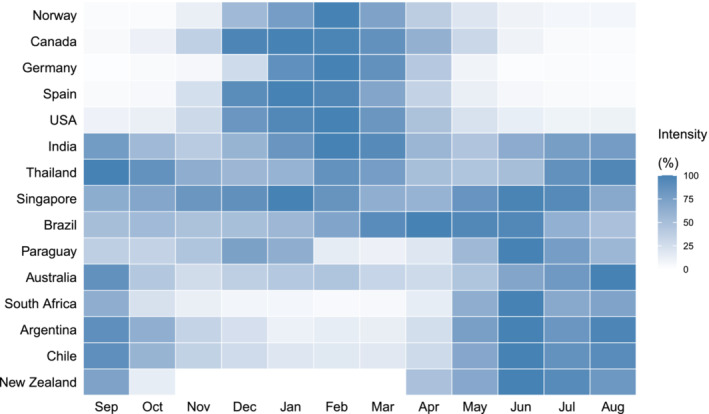
Heatmap of influenza intensity from September 2010 to August 2019 in countries, sorted by the latitude of the country centroid from north to south. The color scale represents the intensity from high (blue) to low (white). Influenza intensity was calculated as the monthly influenza positive rate standardized by the maximum value for that country. The monthly influenza positive rate was calculated as the mean of positive influenza samples each month divided by the mean of samples being tested for the same period. Data source: WHO FluNet database.

The first reason to consider a twice‐annual vaccination strategy is because influenza activity can occur year‐round in some locations (Figure 1). In temperate locations, influenza epidemics generally occur in the winter months, with lowest levels of activity in the summer [7, 8]. Influenza vaccination campaigns therefore take place in the autumn months, for example, September and October in the Northern Hemisphere, so that population immunity will be highest during the winter peak. Influenza seasonality is less predictable in some tropical and subtropical locations [7], making it more challenging to determine the optimal vaccination timing as well as the vaccine composition to use (Northern Hemisphere or Southern Hemisphere). WHO recommends that once the timing of a vaccine program has been decided based on the surveillance data, the most recent vaccine composition available should be used in that program regardless of the hemisphere where the country is located [9, 10].

Several countries or cities located in the Northern Hemisphere have imported and distributed influenza vaccines with the Southern Hemisphere formulation. India, located in the Northern Hemisphere, has influenza peaks from June to August in its southern cities [11, 12], and the National Centre for Disease Control in India recommends using the Southern Hemisphere formulation when the circulating virus matches with the Southern Hemisphere composition [13]. In Thailand, influenza vaccine is available between April and May with the Southern Hemisphere formulation based on its influenza seasonality [14]. Singapore, a tropical country with year‐around influenza transmission and biannual influenza peaks, offers twice‐annual influenza vaccination in situations when the vaccine composition is updated from the latest recommendation [15]. In Hong Kong, concern about a drifted influenza A(H3N2) strain in 2015 led to the one–off use of a spring vaccination dose with the Southern Hemisphere vaccine formulation for older adults, who routinely receive the Northern Hemisphere formulation in the autumn each year [16]. In addition, there are also countries located in the Southern Hemisphere using the Northern Hemisphere formulation [17].

### 2.2 Waning Over Time in Influenza Vaccine Immunogenicity

The second reason to consider twice‐annual vaccination is waning in protection. With earlier availability of influenza vaccines in recent years, even as early as July and August in some Northern Hemisphere locations, it is still typically recommended to offer influenza vaccine during September or October to maintain clinical protection through the winter [18]. Haemagglutination inhibition (HAI) antibody titers, the most widely used laboratory indicator for vaccine‐induced protection, peak at around 3–4 weeks after vaccination and then decay. Some studies have estimated that postvaccination HAI antibody titers and the proportion with antibody titer ≥ 40 (which is established as a protective threshold) decreased significantly at 6 months after vaccination in older adults [19, 20, 21]. Although the duration of antibody response to influenza vaccination should be sufficient to cover a typical influenza season for most temperate locations, annual vaccination might not provide year‐round protection for tropical and subtropical locations (Figure 1).

A second dose may be needed to optimize CD4 T cell‐mediated immunity, which may offer a broader and longer lasting protection against symptomatic influenza, even in the absence of neutralizing antibodies [22, 23]. A study comparing the long‐term duration of humoral and cellular immunity between single and two doses of adjuvanted A(H1N1)pdm09 vaccine found that after two doses of vaccination with an interval of 21 days, influenza‐specific CD4 T cell and memory B cell significantly increased and persisted through 1 year in both younger and older adults [24]. Further research is needed to determine if two doses 6 months apart would have a similar benefit to CD4 T cell‐mediated immunity for older adults as an additional dose.

### 2.3 Waning Over Time in Influenza Vaccine Effectiveness

Declines in influenza vaccine effectiveness (VE) against influenza disease are consistent with observations of declines in vaccine‐induced antibodies after vaccination. Several observational studies have reported waning in VE in preventing influenza disease over time. A test‐negative design study in Singapore estimated that influenza vaccine, when received more than 6 months ago, did not provide protection against laboratory‐confirmed influenza during an outbreak in long‐term care facilities [25]. A multicenter case control study in Europe investigating VE against polymerase chain reaction (PCR)‐confirmed influenza‐like illness from 2010/11 to 2014/15, found that VE against influenza A(H3N2) declined to zero within 4 months after vaccination, whereas waning in VE against influenza A(H1N1) and B strains were not that prominent [26]. Consistent with these findings, data from the US Influenza Vaccine Effectiveness Network from 2011/2012 to 2014/2015 estimated that VE against PCR‐confirmed acute respiratory illness was reduced to low levels after 6 months for influenza A(H3N2), although protection was maintained at higher levels for longer for influenza A(H1N1) and B subtypes [27]. A retrospective cohort study in Singapore estimated that VE against PCR‐confirmed hospitalization was stable in the first 6 months after vaccination but declined afterwards, particularly in older adults [28]. Reduced VE has been observed more frequently for influenza A(H3N2) vaccine strains [29, 30, 31, 32, 33].

### 2.4 Frequent Updates of Vaccine Strains due to Virus Evolution

**FIGURE 2:**
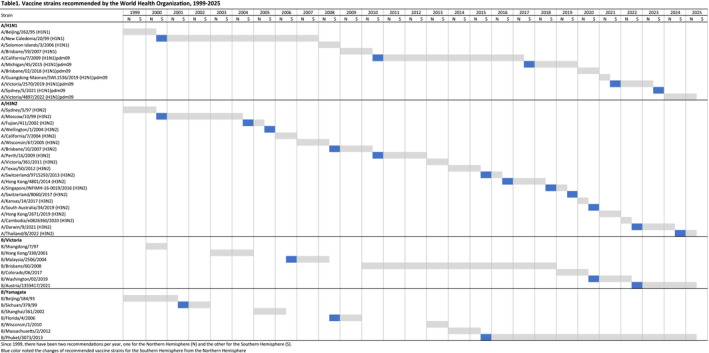
Influenza vaccine strain compositions recommended by WHO from 1999 to 2025 by type and subtype. Blue squares are used to denote the occasions when the Southern Hemisphere vaccine strain was updated from the previous Northern Hemisphere composition. Prior to 2013, only one influenza B strain was recommended each year.

The third reason to consider twice‐annual vaccination is because of the twice‐annual updates of vaccine strains. Because the production of influenza vaccines takes at least 5 to 6 months when the recommended vaccine composition includes updated strains [34], by the time that the updated vaccine is available in the market, the predominant circulating virus strains may have evolved to have antigenic differences from the vaccine strains, affecting VE [28]. The Global Influenza Surveillance and Response System was established by WHO to monitor influenza virus activity and emergence of new strains. Vaccine strain recommendations are issued based on the information available through this surveillance system and other relevant data, and recommendations have been issued twice a year since 1999, in February each year for the forthcoming winters in the Northern Hemisphere and in September for the following winter in the Southern Hemisphere [35]. We reviewed influenza vaccine strain recommendations over a period from 1999/2000 to 2024/2025 and found that approximately half of the strain changes (23/45 changes, 51%) occurred first in the Southern Hemisphere vaccine (Figure 2). This included 60% (12/20) of the updates in influenza A(H3N2) strains specifically.

## 3 Potential Challenges for Twice‐Annual Vaccination

### 3.1 Knowledge Gaps for Clinical Vaccine Effectiveness

There are at least three potential obstacles to implementing a twice‐annual influenza vaccination strategy. First, there is a gap in data on the clinical effectiveness of this strategy. We searched the literature on twice‐annual vaccination and identified one clinical study and one observational study. A randomized controlled study in Singapore compared HAI antibody response and incidence of influenza‐like illness between older adults randomly allocated to annual or twice‐annual vaccination. Both groups were vaccinated at the start of the trial, and after 6 months, one group received an additional dose of influenza vaccine, whereas the other group received a comparator vaccine [36]. Older adults who received a second dose after 6 months had higher influenza A titers at Month 7 and a lower incidence of influenza‐like illness during Months 7–12, compared to those who did not receive the 6‐month dose [36]. An observational study in Hong Kong found that those who received a 2015 Southern Hemisphere influenza vaccine had better humoral and cellular immunity at the start of the 2015/2016 season compared to those who had not received the Southern Hemisphere dose. After the Northern Hemisphere 2015/2016 vaccination, 2015 Southern Hemisphere receipts had similar proportions with postvaccination HAI antibody titer ≥ 40 but lower postvaccination geometric mean titers compared to those who only received that Northern Hemisphere vaccine [16]. The results of these two studies support the potential for additional benefits of twice‐annual vaccination in locations without clear seasonality, although neither of the studies were able to examine whether there were differences in the risk of laboratory‐confirmed influenza between the two groups. Finally, a 5‐year randomized controlled trial comparing the immunogenicity of annual versus twice‐annual vaccination has been underway in Hong Kong since 2016/17 with results anticipated in early 2025 (Clinicaltrials.gov NCT02957890).

### 3.2 Repeat Vaccination Effects

Although some clinical studies have demonstrated the benefits of twice‐annual vaccination in improving immunity, the possibility of increasing the number of vaccinations per year raises questions about potential adverse effects of repeated vaccination, and this is a second potential obstacle to a twice‐annual vaccination strategy. In some years, it has been reported that the incidence of symptomatic laboratory‐confirmed influenza is significantly lower in individuals who have received vaccination for the first time (or at least the first time in recent years) compared to individuals vaccinated in the current season and previous seasons [37, 38, 39]. The mechanisms underlying this effect have not been fully elucidated but reduced immune responses to repeat vaccination likely play a role [40], due to ceiling effects [41], antigenic distance [42], and potentially epitope focusing [43] or antigenic seniority [44].

A recent systematic review and meta‐analysis estimated attenuated VE associated with repeated vaccination by calculating the absolute difference in VE for individuals vaccinated in the current season only and the previous season only compared to both the current and previous season. They found attenuated VE for vaccination in current and previous season compared to current season only, and this attenuated effect was most profound for A(H3N2) subtypes [45]. For repeated vaccination for multiple seasons, a test‐negative design study investigated correlation between VE against laboratory‐confirmed influenza illness and vaccination history in previous 5 years. This study found the highest VE in participants who were not vaccinated in the previous 5 years, moderate VE in those infrequently vaccinated and lower VE in those frequently vaccinated [46]. Consistently, immunogenicity studies also demonstrated that the number of vaccinations received in the previous seasons were associated with attenuated antibody fold rises with a dose–response pattern [47, 48]. Although the current vaccination is less immunogenic for individuals who were vaccinated in the previous seasons compared to those who were not, it remains essential for people to receive influenza vaccine, regardless of their vaccination history, to gain protection during the current season, particularly for those at risk of more severe disease [45, 49].

One of the concerns about twice‐annual vaccination is that reduced immune responses or reduced protection in repeat vaccinees could be accelerated by increasing the vaccination frequency. Repeated vaccination effects have been observed in a study conducted in Hong Kong, showing a reduction in the postvaccination geometric mean titer to the Northern Hemisphere influenza vaccine related to the previous receipt of Southern Hemisphere vaccine 6 months earlier [16]. It remains to be determined whether attenuated effect of repeated vaccination would be more prominent for twice‐annual vaccination than annual vaccination in the longer term. Even if responses are attenuated with repeat vaccination, there might still be an advantage of providing the most updated vaccine strains at 6 month intervals when there is a strain change.

### 3.3 Cost‐Effectiveness and Feasibility

A third potential obstacle relates to the cost and feasibility of twice‐annual influenza vaccination. Regarding feasibility, recent experiences with COVID‐19 vaccines are informative. Although third doses of COVID‐19 vaccination were initially given at 6 months after the second dose, with reasonable uptake of third doses in many locations [50, 51, 52], subsequent COVID‐19 booster dose uptake has been less enthusiastic in many locations [53, 54]. Nonetheless, adding second doses of a vaccine within the same year within existing medical and public health infrastructure proved to ultimately be feasible.

Regarding cost, a twice‐annual vaccination program would probably cost roughly twice that of an annual program. However, the cost‐effectiveness of this new vaccine strategy would depend on its added benefit. Enhanced influenza vaccines are preferentially recommended for older adults in some locations despite costing up to two to three times that of a standard dose vaccine, supported by the estimations that cost savings on healthcare expenditure far exceed the incremental costs of using enhanced influenza vaccine rather than standard‐dose vaccine in older adults [55, 56]. Although delivery costs and administrative costs should also be assessed, it is possible that a twice‐annual vaccination program with standard dose vaccines might have similar overall costs to an annual vaccination program with an enhanced vaccine. A modeling study reported that twice‐annual vaccination may be a more cost‐effective strategy than annual vaccination for older adults in locations with no clear influenza seasonality [57]. Disease burden data are an important input into cost‐effectiveness evaluations. More specific burden estimates than those typically available may be needed for a full evaluation, such as burden estimates for older adults in long‐term residential care or community dwelling older adults with more serious underlying medical conditions including immunocompromising conditions. These groups might be the initial target for a twice‐annual program, given their higher risk of severe influenza. In addition, attempts to balance the costs and benefits of a midyear vaccination dose could be considered and evaluated, for example, a flexible approach using midyear doses only when there is a significant vaccine update.

## 4 Discussion and Conclusions

The concept of twice‐annual vaccination might have relevance not only to tropical and subtropical areas but in temperate locations as well. A midseason vaccination dose could potentially provide additional protection to residents of long‐term care or other high risk individuals [58]. In this case, the vaccine would be the same formulation, and the timing of the additional dose would likely be 3 months rather than 6 months after the usual autumn dose to provide increased protection during a late season. In addition, we have focused here on the arguments for or against twice‐annual administration of standard dose vaccines, but similar arguments might be applied to twice‐annual use of cell‐based vaccines or enhanced influenza vaccines. However, we currently have very limited data on repeat vaccination with cell‐based or enhanced vaccines and twice‐annual vaccination with these products warrant further investigation.

We have outlined above the rationale for considering a twice‐annual vaccination strategy. The first reason for considering twice‐annual vaccination is because subtropical and tropical locations can experience prolonged influenza seasons. The second reason is because the protection provided by annual influenza vaccination wanes substantially after 6 months and twice‐annual vaccination could mitigate this. The third reason is that receipt of the most recent vaccine strains every 6 months could improve protection for the second half of the year, particularly when vaccine strains have been updated. The three obstacles discussed above include knowledge gaps on the clinical effectiveness of twice‐annual vaccination, whether there might be any potential negative consequences of more frequent influenza vaccination due to “repeat vaccination effects” and potential concerns about the feasibility or cost of a twice‐annual program. At present, there does not seem to be sufficient evidence to allow an informed policy decision, but we argue that there is sufficient rationale as well as sufficiently supportive preliminary data that twice‐annual influenza vaccination deserves further consideration as a potential vaccination strategy.

## Author Contributions

**Shuyi Zhong:** conceptualization, writing – original draft, visualization. **Mark G. Thompson:** writing – review and editing, conceptualization. **Benjamin J. Cowling:** conceptualization, writing – review and editing, funding acquisition, supervision.

## Conflicts of Interest

MGT has consulted for Novavax and CSL Seqirus. BJC has consulted for AstraZeneca, Fosun Pharma, GlaxoSmithKline, Haleon, Moderna, Novavax, Pfizer, Roche, and Sanofi Pasteur.

### Peer Review

The peer review history for this article is available at https://www.webofscience.com/api/gateway/wos/peer‐review/10.1111/irv.70052.

## Data Availability

No new data were generated or analyzed in this study, and data sharing is not applicable to this article.
